# Geochemical Fractionation and Assessment of Probabilistic Ecological Risk of Potential Toxic Elements in Sediments Using Monte Carlo Simulations

**DOI:** 10.3390/molecules24112145

**Published:** 2019-06-06

**Authors:** Sanja Sakan, Nenad Sakan, Aleksandar Popović, Sandra Škrivanj, Dragana Đorđević

**Affiliations:** 1 Centre of Excellence in Environmental Chemistry and Engineering–ICTM, University of Belgrade, Njegoševa 12, 11158 Belgrade, Serbia; 2 Institute of Physics, University of Belgrade, Pregrevica 118, 11080 Zemun, Serbia; nsakan@ipb.ac.rs; 3 Faculty of Chemistry, University of Belgrade, Studentski trg 12-16, 11000 Belgrade, Serbia; apopovic@chem.bg.ac.rs (A.P.); sandra_skrivanj@chem.bg.ac.rs (S.Š.)

**Keywords:** geochemical fractionation, probabilistic ecological risk, Monte Carlo simulation, toxic elements, sediments, hierarchical clustering

## Abstract

The need for further research into potentially toxic elements in Serbian rivers led to an investigation of distributions, sources, and ecological risks in a sample base of sediments from 15 rivers. The analyses were carried out through both experimental and theoretical methods. Geochemical fractionation of Cd, Co, Cr, Cu, Fe, Mn, Ni, Pb, As, V, and Zn in sediments was studied using a sequential extraction procedure. Both a Håkanson risk index (RI) and a Monte Carlo simulation (MCS) were used in order to estimate ecological risk, applying the probability distribution of RI values instead of single-point calculations. In order to both further the development of the used method and include additional processes, software for the simulations was developed instead of using proprietary solutions. Metal fractionation showed high percentage recoveries of Cd, Cr, Co, Cu, Fe, Ni, and V in residual fractions. The high content of Pb, Mn, and Zn in mobile fractions might cause serious environmental concerns. In some localities, Cu and Cd could be problematic elements, since their mobility was high. An environmental assessment based on the described criteria provided risk levels varying from low to median (mainly contributed by Cd and Cu).

## 1. Introduction

As an essential part of the overall ecological system, water is a subject and special responsibility in terms of improving the quality and sustainability of the quantity and needs of future generations. Due to intensive technological and industrial development, a large amount of harmful and toxic substances is polluting rivers and river sediments and is causing environmental damage. Among these substances, pollution by toxic and potentially toxic elements (PTEs) is a major problem due to their ubiquity coinciding with their toxicity and persistence [1]. River sediment quality is estimated by the presence of the sought toxic elements in fluvial sediments and fine suspended particles, since the solubility of the investigated PTEs in water is low under normal conditions [2].

When PTEs are found in an aquatic environment, they can be accumulated in sediment. River sediment is characteristic of the great complexity and diversity of its composition. The binding capacity for these elements depends primarily on the composition of the original rock and climatic conditions. Reactivity or PTE mobility, and therefore potential toxicity, depends on the substrate to which it is related and the strength of the bound. The biological activity and chemical reactions in a water column can mobilize some of the toxic elements from sediment, and they can be carried out down the stream [3]. From the point of view of the environment, it is important to determine under what conditions PTEs can be released from sediment. 

PTEs can be bounded in various matrices in different ways: occluded in amorphous materials; adsorbed on clay surfaces or iron/manganese oxyhydroxides; presenting in lattice of secondary minerals like carbonates, sulfates or oxides; complexed with organic matter (OM) or lattice of primary minerals such as silicates [4]. The strength of the element’s binding determines its bioavailability as well as the risk associated with its presence in the investigated system. The strength values can, therefore, give a clear indication of sediment reactivity, which in turn allows an assessment of the risk connected with the presence of metals in the environment [5]. In an unaffected ecosystem, PTEs are almost immobile, as they are bound to silicates and minerals, but under the influence of human activities, these elements may be found in other labile forms, such as oxides, carbonates, hydroxides, and sulfides [1].

Extensive studies of different extraction methods for environmental samples have been carried out [6,7,8,9,10]. Since metals are bound into different chemical forms associated with a matrix, an analysis requires the application of sequential extraction methods [11]. Sequential extraction (SE) is an important and widely applied tool for gathering information on the potential mobility (hence, potential bioavailability and toxicity) of potentially toxic metals in the environment [12].

Different evaluation methods are used to determine the degree of contamination for sediments using numerous pollution indices [3,7,9,13,14,15,16]. Most current ecological risk assessment of sediments, including RI method, assume and combine a series of average, conservative and worst case values to derive a conservative point estimate of risk [17]. An evaluation of complex situations involving random behavior can be carried out with the help of Monte Carlo methods, which can help reduce uncertainty in estimating future outcomes in areas such as risk assessment or actuarial analyses [18]. This technique provides a quantitative way to obtain probability distributions for risks within the validity of the assessment model and provides more information for decision-making [17].

Serbia is very rich in water resources, not only due to the Sava, Danube, and Tisa rivers, but also due to mountain springs. Water pollution is a significant problem in Serbia and comes mainly from outdated technology, a lack of pollution abatement installations, inadequate storage and disposal of byproducts, untreated industrial and municipal wastewater, drainage water from agriculture, etc. Considering the large pollution of many river flows in Serbia, research related to their quality in Serbia are very important. First of all, new approaches and methods should be developed for applications with which it would be possible to monitor the quality of water.

A thorough investigation of the mobility of PTEs in river sediments was carried out within this manuscript by applying experimental and theoretical methods. This investigation was conducted to (i) quantify and assess the spatial variations of the studied elements, Cd, Co, Cr, Cu, Fe, Mn, Ni, Pb, V, and Zn, in river sediments collected from 15 rivers in Serbia; (ii) evaluate the potential mobility of elements in various fractions of sediments using a modified Tessier’s sequential extraction method; (iii) determine the contamination factor to assess the degree of toxic element risk to the environment in relation to retention time; (iv) perform an ecological risk assessment of potentially toxic elements using a Monte Carlo simulation (MCS); and (v) determine the interrelations and similarities of the extracted element contents in each extraction step using cluster analysis. 

To our knowledge, this is the first report evaluating the probabilistic ecological risk of potentially toxic elements in Serbian river basins by performing a Monte Carlo simulation. The information presented as a result of the carried-out study could be directly used in planning appropriate strategies for the environmental management of these drainage basins.

## 2. Materials and Methods

### 2.1. Studied Area

The territory of Serbia is characterized by a varied lithographic composition. Several geotectonic compositions can be singled out within this territory: the Pannonian Basin, Inner Dinarides, Vardar Zone, Serbo-Macedonian Massif, Carpatho-Balkanides, and Dacian Basin. Masses of karstified limestone are the main water-bearing media in morphologically broken-up regions (the Dinarides and Carpatho-Balkanides). Water-bearing media in the Vardar zone consist almost exclusively of rocks with fracture porosity, and water-bearing media of young depressions (the Pannonian and Dacian basins, as well as depressions within the Serbo-Macedonian Massif) are represented by alluvial formations and Neogene lacustrine sediments, i.e., water-bearing media with intergranular porosity [19].

The river network in the region of Serbia is relatively dense. The greatest part of Serbian territory belongs to the Danube drainage basin. The most important tributaries of the Danube in Serbia are the Tisa, Sava, and Great Morava rivers [20]. 

### 2.2. Sampling Sites

**Figure 1:**
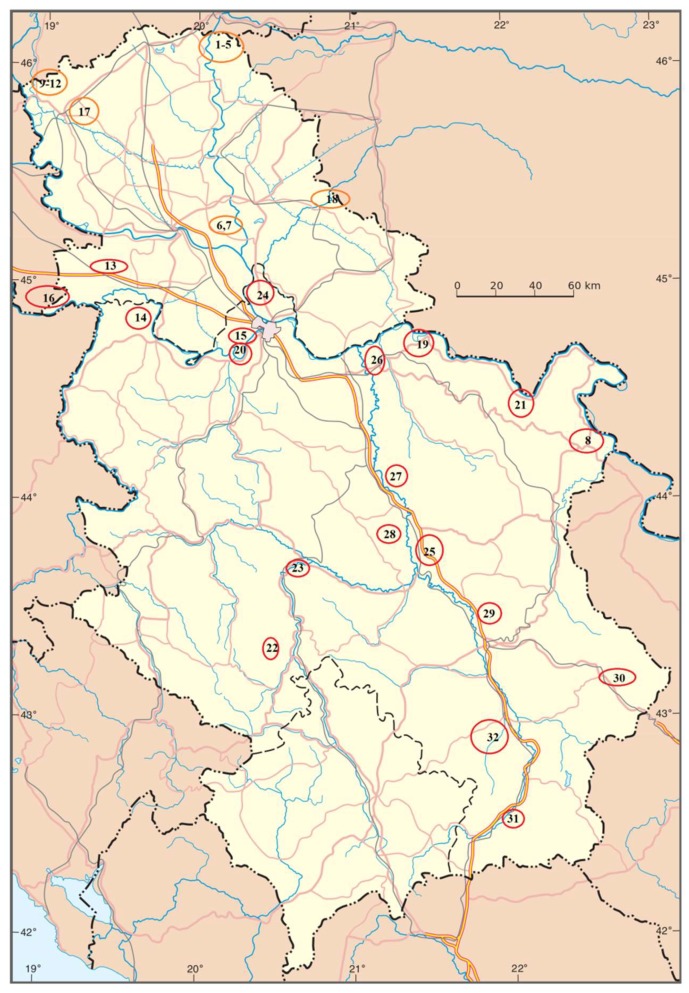
Locations of sampling sites.

Thirty-two samples of river sediment from 15 rivers in Serbia were collected for this research (Table 1, Figure 1): the Danube, the Sava, the Tisa, the Ibar, the Great Morava, the West Morava, the South Morava, the Nišava, the Tamiš, the Danube–Tisa–Danube Canal (DTD, Vrbas), the Topčiderska River, the Porečka River, the Kolubara, the Pek, and the Toplica. For the larger rivers, sampling was conducted at several locations (Figure 1). The sediment samples were stored at 4 °C. The contents of the micro- and macroelements were determined from the granulometric fraction <63 μm of the bottom sediment [21].

### 2.3. Chemical and Data Analysis

Geochemical fractionation was conducted through a modification of Tessier’s sequential extraction, which included five phases: F1 (“ion-exchangeable”), which was adsorbed and water-soluble metal forms and much less metal bound to carbonate (1 M CH_3_COO(NH_4_)); F2, which was metal bound to carbonate and easily reducible species (0.01 M HCl and 0.1 M NH_2_OH·HCl); F3, which was metal bound to moderately reducible phases or the Fe oxide fraction (0.2 M H_2_C_2_O_4_ and 0.2 M (NH_4_)_2_C_2_O_4_); F4, which was organic matter and sulfides (30% H_2_O_2_ adjusted to pH 2 with HNO_3_, followed by extraction with 3.2 M CH_3_COO(NH_4_)); and F5, which was a “residual” fraction (aqua regia). A description of the procedure for fractions 1–4 is shown in Reference [5], and the fifth fraction is in Reference [12]. Samples were microwave-digested to determine the total contents of the studied elements [14,21]. The concentrations of elements from the extracts were determined by ICP/OES (inductively coupled plasma atomic emission spectrometry; iCAP-6500 Duo, Thermo Scientific, Cambridge, UK). 

Quality control, accuracy, and the precision of the measurement and concentration values were performed using a certified reference material, BCR-143R. The measured values were in excellent agreement with the certified values of the BCR 143 reference materials (the accuracy ranged from 81.5% to 114%). Precision was expressed as the relative standard deviation. The relative standard deviations of the means of duplicate measurements were less than 10%.

Descriptive statistics and a hierarchical cluster analysis (HCA) were carried out using SPSS version 21 for Windows.

Determination of the toxic element contamination factor is an important aspect that indicates the degree of toxic element risks to the environment in relation to its retention time [3]. These factors are defined as the sum of element contents in the mobile phases (nonresidual phases) of the sample divided by the residual phase content (*C_f_* = Σ (step 1 + 2 + 3 + 4)/residual, step 5). The lower the *C_f_* value is, the higher the relative metal retention is [13].

### 2.4. Ecological Risk Analysis

Håkanson’s method could be used to evaluate the potential ecological risk of metal contaminants in sediments [22]. A potential ecological risk index (RI) shows the potential ecological risk caused by various pollutants in the environment [23,24]. According to Håkanson’s method, the RI of metal contaminants in sediments can be calculated using the following equation:

(1) $$\mathrm{RI}=\sum_{i=1}^{m}{E_{r}}^{i},$$

where *E_r_^i^* = *T_r_^i^* · *C_f_^i^* and *C_f_^i^* = *C^i^*/*C^i^_n_*.

*RI* are calculated as the sum of all risk factors for heavy metals in sediments, *E_r_^i^* is the potential ecological risk for a single factor, *T_r_^i^* is the toxic response factor for a given metal, *C_f_^i^* is the contamination factor, *C^i^* is the measure concentration of metals in sediment, and *C^i^_n_* is the reference value for metals.

### 2.5. Monte Carlo Simulation

A Monte Carlo analysis based on mathematical statistics and probability theory was used to assess model uncertainty through random sampling of a probability distribution for each variable [25]. In this manuscript, instead of Håkanson’s RI, the probabilistic distribution of the RI was calculated using a Monte Carlo simulation to randomly sample values from the distribution of exposure concentrations [24].

The Monte Carlo method has been proven as a modeling procedure for stochastic processes, and its application originates from the nuclear age era [26], when it was usually applied to particle transport problems. As such, it is also an applicable, if not unavoidable, tool for risk assessment. As could be seen from Qu et al. [17] and Wu et al. [27], the Monte Carlo method is very applicable in heavy metal pollution risk assessment. Based on their work, we developed our software, which is written in Qt [28], and a random number generator produces a normal distribution with long-term repeatability. The program used was tested on several models, and as a final test the results from Qu et al. [17] and Wu et al. [27] were reproduced in their entirety based on input data. It is expected that the use of our specific software could lead us to more easily extend its capabilities based on the current need in research.

## 3. Results and Discussion

### 3.1. Distribution by Fraction of Studied Elements

**Figure 2:**
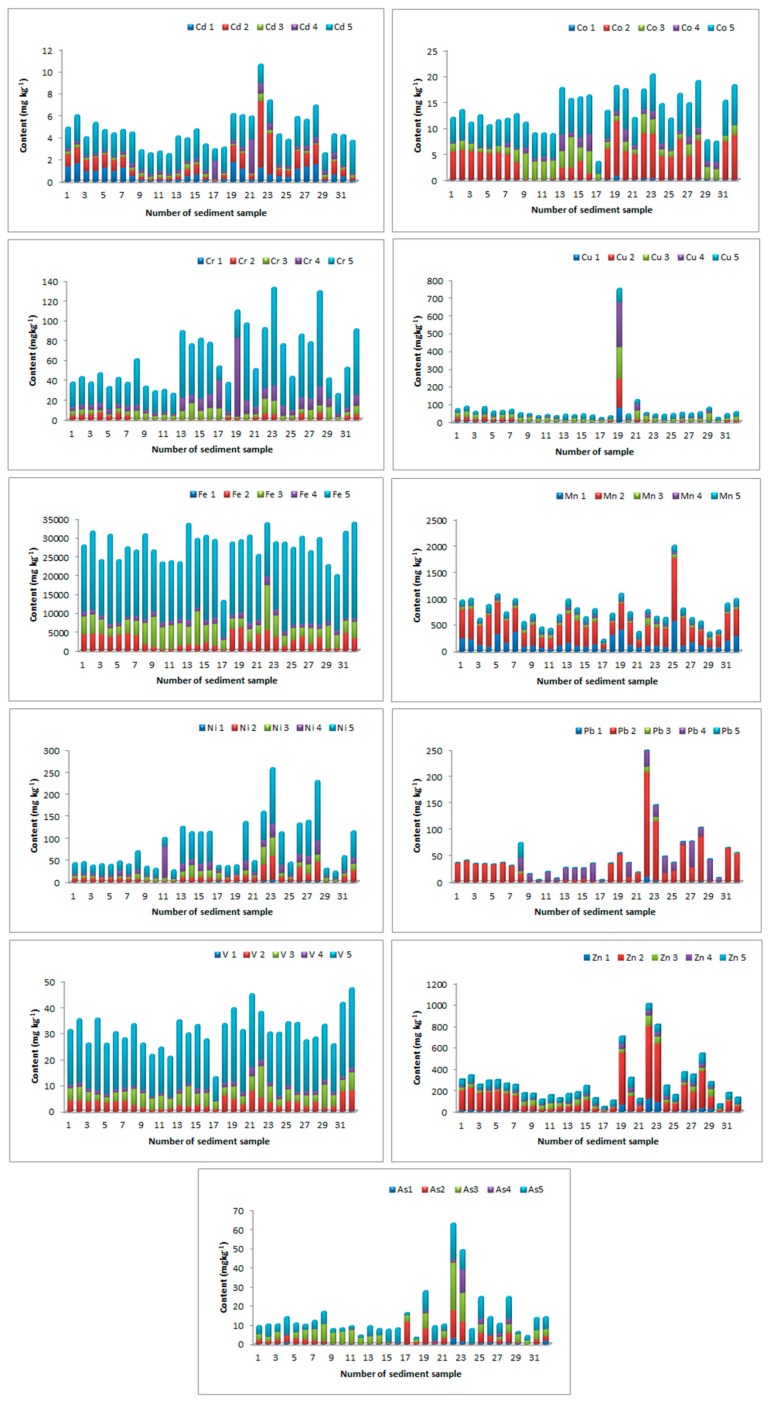
Distribution of Cd, Cr, Cu, Fe, Mn, Ni, Pb, V, Zn, and as by fraction and sampling site. *E1: “ion-exchangeable”; E2: metal bound to carbonate and easily reducible species; E3: the Fe oxide fraction; E4: organic matter and sulfides; E5: the “residual” fraction.

A sequential extraction procedure was applied to fractionate the studied elements within sediments. The percentage element distribution by fraction was calculated as the average content of the extracted element in each fraction with respect to the total extracted element content (Table 2). The residual fractions of the sediment were the dominant ones for Cd, Cr, Co, Cu, Fe, Ni, As, and V, as shown in Figure 2 and Table 2. This fact indicated that crystal forms of iron oxides as well as aluminosilicates were a significant substrate of the studied elements.

Those residual fractions were relatively stable and did not show significant transformations in various conditions: Namely, the metals still remained in sediment. Residual fractions of both geogenic and anthropogenic origin represented the more stable metal forms, and their influence on the ecological system was much less than the others under normal conditions. The dominant fractions for the extraction of Co, As, and V were F5, F2, and F3. This distribution indicated the fact that Fe oxide of different degrees of crystallinity was the most significant for the binding of Co, As, and V. The change in oxidation state of Fe and Mn could lead to the release of the elements that were retained in this form, and this accidental situation could lead to a long-term source of contamination. The Mn, Pb, and Zn are extracted significantly in mobile, the second fractions. This distribution indicated that carbonates and oxide fractions, principally mobile fractions, were the most significant for the binding of these elements. As shown in Tessier et al. [29], metals bound in carbonate and exchangeable, Fe-Mn oxide, and organic fractions are the most likely to mobilize from the sediments if oxygen or the geochemical conditions change in the surface water and hence are more available for the food chain. The fractions bound to Mn oxides and organic materials were reviewed as the most important components in sediments for metal binding, and they represented a potentially mobile component under changing conditions.

An organic fraction may be associated with various forms of organic material, such as living organisms, detritus, or coatings on mineral particles, through complexation or a bioaccumulation process. This kind of metal can exist in sediment for longer periods, and can also be released with OM decomposition. An exchangeable fraction, referring to metals directly adsorbing on sediments, was not significant for elements in the studied sediments. However, in some localities, it was possible to observe a substantial proportion of Cd and Cu extracted in the mobile, first, and second fractions (Figure 2). Since exchangeable and carbonate bound fractions (weakly bound forms) are partially introduced through anthropogenic intrusions [10], it can be assumed that in some localities there existed local anthropogenic sources of Cd and Cu.

In the current investigation, Pb, Mn, and Zn were noted to be highly mobile, and their high concentration in the mobile fractions might cause serious environmental concerns. In addition, in some localities, Cu and Cd could be problematic elements, since their mobility was high.

### 3.2. Distribution of Elements by Sampling Site

**Figure 3:**
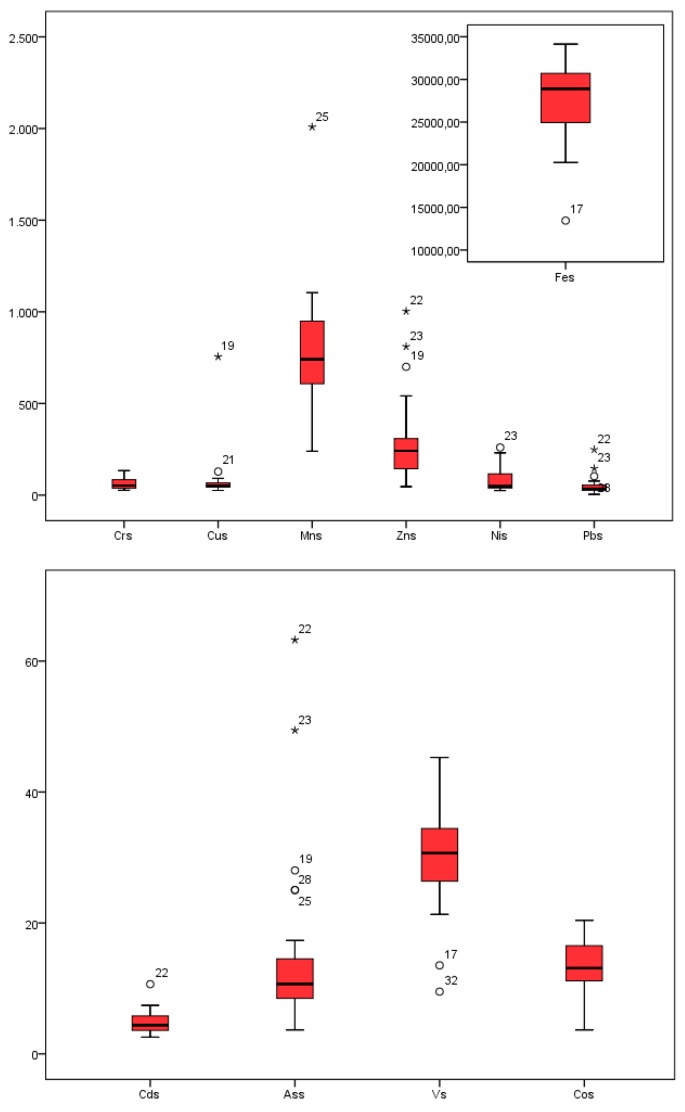
Box plot diagrams of Cd, Cr, Cu, Fe, Mn, Ni, Pb, V, As, and Zn.

The total content of elements after digestion with aqua regia, the guideline values of Serbia, background values, and earth crust values of elements in the studied sediments are presented in Table 3. The distribution of elements by sampling site is given in Figure 2. A comparison of the quantitative distribution of the contents of the various elements in the sediments is shown in a boxplot (Figure 3).

Outlier and extreme values of the element contents suggested that the most critical sites in terms of metal contamination were observed in West Morava (Pb), Ibar (Cd, Zn, Pb, and Ni), the Porečka River (Cu), Pek (Cu, Zn), and South Morava (Mn). The maximum content of Cd, Cr, Cu, Ni, Pb, and Zn exceeded the standard values given by Serbian guidelines. Permanent and accidental pollution from industrial plants and mines that are located in the basins of these rivers, alongside agricultural use of manganese-containing products such as fertilizers and fungicides, were the main cause of pollution in these rivers [32]. Given the existence and position of a Cu mine in Majdanpek close to the source of the Pek River, it is justifiable to assume that the origin of the Cu was associated with this mine [21].

As the greatest tributary of the West Morava, the Ibar River is affected with lead and zinc pollution, since in this area are a vast number of production and manufacturing plants of the mining-metallurgical system, Trepča-nine lead and zinc mines, three flotations, two of metallurgy, the chemical industry, and a battery factory [33].

### 3.3. Cluster Analysis (CA)

**Figure 4:**
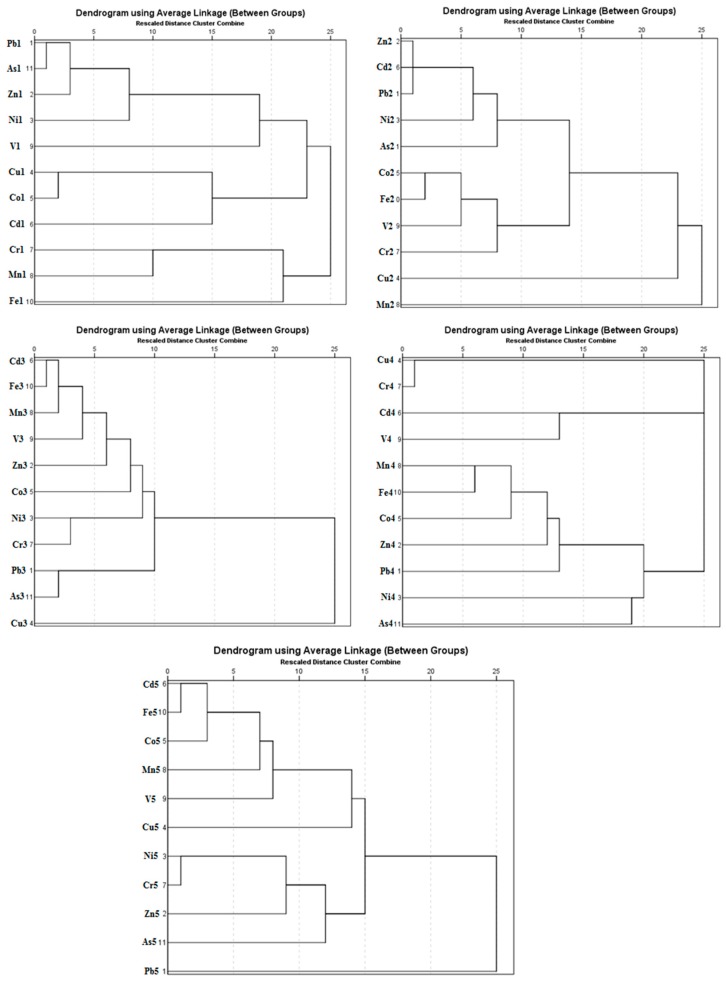
Presentation of the cluster analysis (CA) for the different steps recovered in the sequential extraction procedure (SEP). The number shown next to the element represents a fraction of the sequential extraction.

A cluster analysis (R-mode, Pearson method) was carried out by using the contents of the elements extracted in the different fractions. The results of the cluster analysis by fraction of sequential extraction are shown in Figure 4.

Fraction F1: The results for the F1 fraction distinguished the elements into three groups, which were formed by mixing different mineralogical species. Group 1 was characterized by elements Pb1, As1, Zn1, and Ni1, mainly due to the presence of secondary carbonate minerals that were partially destroyed in this fraction. Group 2 was constituted by Cu1, Co1, and Cd1, which did not show a clear association and could form a mixing group where different mineral species such as hydrated oxides of iron and manganese, humic acids, or sulfates could present their substrates. Group 3 was constituted by Cr1, Mn1, V1, and Fe1, which could be attributed to the important role proposed for Fe and Mn oxyhydroxides in the retention of elements in this fraction.

Fraction F2: Fraction F2 could be differentiated into three groups with a high association level. Group 1 was characterized by elements such as Cd2, Zn2, Pb2, Ni2, and As2 due to the presence of carbonate minerals, and these had a high-potential capacity to collect and retain metallic elements on the surface [2]. Group 2 was constituted by Fe2, Co2, V2, and Cr2, which may indicate Co, V, and Cr were associated with hydrated oxides of iron and that Group 3 was associated with Cu3 and Mn3, which may indicate an association of Cu with hydrated oxides of manganese.

Fraction F3: An analysis of the F3 fraction did not show as clear a differentiation as the other fractions. Cd3, Fe3, Mn3, V3, Zn3, Co3, Cr3, Ni3, Pb3, and As3 made one group and isolated only Cu3. This distribution was the result of the dissolution of the partially crystalline and amorphous iron oxide forms at this stage, as they are important substrates of those elements. A possible cause for the separation of Cu3 as a special group was probably the different origin of this element with respect to the other elements.

Fraction F4: Fraction F4 could be differentiated into two groups with a high association level. Group 1 was characterized by elements Cr4, Cu4, Cd4, and V4, mainly due to associations of these elements with organic matter. Group 2 was constituted by Fe4, Mn4, Co4, Zn4, Pb4, Ni4, and As4, since these elements are associated with sulfides. As and S may form very insoluble compounds, such as arsenopyrite [34].

Fraction F5: An analysis of the residual fraction showed a differentiation of the elements into three groups. The first group consisted of Cd5, Fe5, Co5, Mn5, V5, and Cu5, where major parts of the elements were related to the presence of crystalline Fe oxides. The second group was composed of Cr5, Ni5, and Zn5, which are mainly related to the presence of silicates; and the third group was isolated Pb5 and As5, which are recognized as having an affinity with sulfides or secondary sulfates (arsenic–lead sulfide minerals). As shown in Noguchi and Nakagawa [34], the following lead–arsenic sulfide minerals are known: Jordanite 4PbS·As_2_S_3_, gratonite 9PbS·As_2_S_3_, guitermanite 1OPbS·3As_2_S_3_, dufrenoysite 2PbS·As_2_S_3_, rathite 13PbS·9As_2_S_3_, baumhauerite 4PbS·3As_2_S_3_, liveingite 5PbS· 4As_2_S_3_, and sartorite PbS·As_2_S_3_.

### 3.4. Contamination Factors

The individual contamination factor (Cf) of elements was used to estimate the relative retention time of heavy metals retained in the sediment. Higher Cf values demonstrated a lower retention time and a higher risk to the environment. In this research, contamination factors were calculated for Cu, Cr, Pb, Cd, Ni, Co, Fe, Mn, Zn, As, and V in the sediments at all stations.

**Figure 5:**
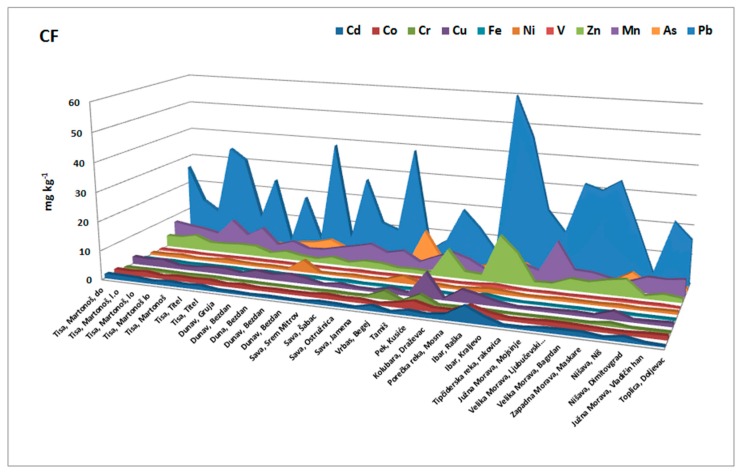
Contamination factor for each element in the investigated sediments.

The obtained results (Figure 5) showed that relative metal retention was not the same for all of the elements. The calculated factors showed the highest Cfs for Pb, Zn, Mn, Cu, and Cd and showed their ability to be released from Serbian river sediments; whereas As, Co, Cr, Fe, Ni, and V showed the lowest mobility and hence the ability to be released. The combined risk of Cd, Zn, Cu, and Pb, the great contributors to highly mobile fractions, was a consequence of their large concentrations, toxicity, and mobility, and as such this presents an increased possible risk of these metals to the environment. The highest contamination factors were obtained for Mn, Zn, and Pb in the sediment samples obtained from Pek and Ibar sediments (locations 19 and 22). It can be concluded that Fe and V had a high relative metal retention with respect to the other elements.

### 3.5. Ecological Risk Assessment of Potentially Toxic Elements Using the Monte Carlo Simulation 

**Figure 6:**
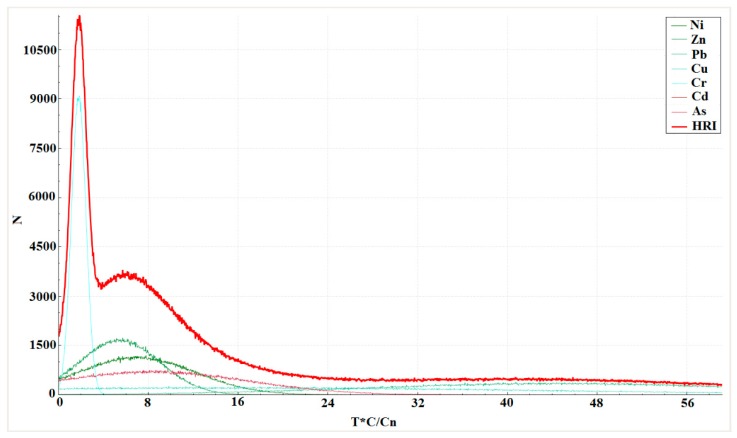
Distribution curve and exceedance probability curves of the risk index (RI) and total ecological risk comprehensive index HRI based on a Monte Carlo simulation run 100,000 times. Local backgrounds are the reference values for the calculation of *E_r_*.

A distribution curve of *E_r_^i^* and HRI (Total ecological risk comprehensive index) values is shown in Figure 6. The probability that ecological risk appeared at different risk levels with reference to a risk level classification standard was analyzed, as shown in Table 4.

The risks of Zn, Pb, and Cr were all low. The risks for Ni and As were also low (Ni 99.07% and 99.99% for As). The Monte Carlo simulations indicated that Cu and Cd posed a relatively high ecological risk in the studied areas, from a low to median risk. These results suggest that Cd and Cu are the most important risk factors in Serbian river basins (especially Cd), given that the probability of a median risk level was 31.70%. The obtained results were in line with the results of the sequential extractions, calculated contamination factors, and box plot method results. The results of the Monte Carlo simulations indicate that mobility, in addition to the high content of an element, is very important in risk assessment.

As shown in Table 5, the probability of the HRI values being at a low risk level was 100%, i.e., the total ecological risk level of heavy metal pollutants in the sediments of rivers in Serbia.

## 4. Conclusions

Content, spatial distribution, and distribution by fraction, as well as an ecological risk assessment of elements (Cd, Co, Cr, Cu, Fe, Mn, Ni, Pb, V, As, and Zn), were studied in surface sediments from 15 rivers in Serbia. The study demonstrated that the sediments contained significant contents of elements. The maximum content of Cd, Cr, Cu, Ni, Pb, and Zn exceeded the standard values given by Serbian guidelines. Outlier and extreme values of element contents were observed in West Morava (Pb), Ibar (Cd, Zn, Pb, and Ni), the Porečka River (Cu), Pek (Cu, Zn), and South Morava (Mn), suggesting that these were the most critical sites in terms of metal contamination.

Among the studied elements, Cd, Cr, Co, Cu, Fe, Ni, As, and V showed dominant levels in residual fractions of the sediments. Mn, Pb, and Zn were extracted significantly in mobile and second fractions. This distribution indicated that carbonates and oxide fractions, principally mobile fractions, were the most significant for the binding of these elements. The combined effects of a high content of Cd, Zn, Cu, and Pb and their high mobility potential present an increased possible risk of these metals to the environment.

Since a high percentage of Cr, Co, Cu, V, and As was extracted in the third extraction stage, it is necessary to emphasize the importance of extraction with an oxalate reagent, considering that poorly crystallized Fe oxide represented a significant substrate of the examined elements. Although this step is not included in many sequential extraction procedures (nor in the sequential extraction procedure proposed by the Community Bureau of Reference (BCR) standard procedure), the results obtained in this study suggest the need for the addition of oxalate reagents in SE procedures, especially when it comes to river sediment as substrate. The developed MCS software gave an opportunity for its further improvement in order to describe complex mathematical models of stochastic systems. The goal for the development of our software is to have the ability to follow both the experimental basis and the theoretical modeling developments of risk pollution assessments. In its current state, it is a good tool for risk pollution assessments, and work on improving its modeling is in progress.

## Figures and Tables

**Table 1:** Sediment samples: Number, river, and sampling site.

Sample	River and Sampling Site
1	Tisa (Martonoš)
2	Tisa (Martonoš)
3	Tisa (Martonoš)
4	Tisa (Martonoš)
5	Tisa (Martonoš)
6	Tisa (Titel)
7	Tisa (Titel)
8	Danube (Gruja)
9	Danube (Bezdan)
10	Danube (Bezdan)
11	Danube (Bezdan)
12	Danube (Bezdan)
13	Sava (Sremska Mitrovica)
14	Sava (Šabac)
15	Sava (Ostružnica)
16	Sava (Jamena)
17	DTD canal (Begej, Vrbas)
18	Tamiš (JašaTomić)
19	Pek (Kusiće)
20	Kolubara (Draževac)
21	Porečka river (Mosna)
22	Ibar (Raška)
23	Ibar (Kraljevo)
24	Topčiderskariver (Rakovica)
25	South Morava (Mojsinje)
26	Great Morava (Ljubičevski most)
27	Great Morava (Bagrdan)
28	West Morava (Maskare)
29	Nišava (Niš)
30	Nišava (Dimitrovgrad)
31	South Morava (Vladičin Han)
32	Toplica (Doljevac)

**Table 2:** Distribution of metals in the fractions of the sequential extraction.

Element	Distribution by Fraction
Cd	F5 > F2 > F1 > F4 > F3
Cr	F5 > F4 > F3 > F2 > F1
Co	F5 > F2 > F3 > F4 > F1
Cu	F5 ≈ F3 > F4 ≈ F2 > F1
Fe	F5 > F3 > F4 > F2 > F1
Mn	F2 > F1 > F5 > F4 > F3
Ni	F5 > F4 ≈ F2 ≈ F3 > F1
Pb	F2 > F4 > F3 ≈ F5 ≈ F1
Zn	F2 > F5 > F4 > F3 > F1
V	F5 > F3 > F2 > F4 > F1
As	F5 > F3 > F2 > F4 > F1

F1: ion-exchangeable; F2: metal bound to carbonate and easily reducible species; F3: the Fe oxide fraction; F4: organic matter and sulfides; F5: the “residual” fraction.

**Table 3:** Comparison of the total element contents in surface river sediments to the guideline values of Serbia, earth crust values, and background values (mg kg^−1^).

	Cd	Co	Cr	Cu	Fe	Mn	Ni	Pb	V	Zn	As
Min	1.28	8.22	59.8	11.5	24,556	648	33.2	57.8	60.4	66.6	3.67
Max	10.5	36.2	230	870	62,800	3688	274	318	149	1095	63.2
Mean	4.82	22.0	113	78.5	44,177	1399	77.8	132	111	353	14.7
Guideline v. ^1^	3	/	100	100	/	/	50	100	/	300	25
Earth crust ^2^	0.13	18	83	47	46,500	1000	58	16	90	83	1.7
Background v. ^3^	1.28	8.22	62.1	11.5	24,556	648		57.8	66.4	66.6	
Toxic response f. ^4^	30	/	2	5	/	/	5	5	/	1	10

^1^ The guideline values of Serbia [30]; ^2^ earth crust values [31]; ^3^ background values of the studied sediments; ^4^ toxic response factors. Here, v.: Values; f.: Factors.

**Table 4:** Ecological risk analysis results of each heavy metal pollutant.

Value of *E_r_^i^*	Risk Level			Probability	(%)			
		Ni	Zn	Pb	Cu	Cr	Cd	As
*E_r_^i^* < 40	Low	99.97	100	100	45.33	100	16.24	99.99
40 ≤ *E_r_^i^* < 80	Lower	0.03	0	0	42.29	0	52.06	0.01
80 ≤ *E_r_^i^* < 160	Median	0	0	0	12.37	0	31.70	0
160 ≤ *E_r_^i^* < 320	High	0	0	0	0	0	0	0
*E_r_^i^* ≥ 320	Extremely high	0	0	0	0	0	0	0

**Table 5:** Total ecological risk analysis results of the rivers.

HRI Value	Risk Level	Probability (%)
HRI < 150	Low	100
150 ≤ HRI < 300	Lower	0
300 ≤ HRI < 600	Median	0
HRI ≥ 600	High	0
